# The expansion of the role of nurse prescribing in intensive care units in the healthcare system of Iran: a qualitative content analysis

**DOI:** 10.25122/jml-2021-0188

**Published:** 2022-02

**Authors:** Azam Naderi, Abbas Abbaszadeh, Marzieh Pazokian, Camelia Rohani, Rostam Jalali

**Affiliations:** 1. School of Nursing and Midwifery, Shahid Beheshti University of Medical Sciences, Tehran, Iran; 2. Department of Medical-Surgical Nursing, School of Nursing and Midwifery, Shahid Beheshti University of Medical Sciences, Tehran, Iran; 3. Community Health Nursing Department, School of Nursing and Midwifery, Shahid Beheshti University of Medical Sciences, Tehran, Iran; 4. Department of Health Care Sciences, Palliative Care Center, Marie Cederschiöld Högskola, Ersta Sköndal Bräcke University College, Stockholm, Sweden; 5. Department of Medical-Surgical Nursing, School of Nursing and Midwifery, Kermanshah University of Medical Sciences, Kermanshah, Iran

**Keywords:** role expansion, Medicine, ICU, qualitative study, nurse prescribing

## Abstract

Intensive Care Unit (ICU) nurses prescribe medication for patients in many countries. However, there is still no evidence on the legitimacy of nurse prescribing roles in the healthcare system of Iran. This qualitative study with 30 experts was conducted to explore the experiences regarding the expanding role of prescribing medication by the ICU nurses. Data were collected through 31 individual semi-structured interviews and analyzed using the conventional content analysis method by MAXQDA 10. One major theme, “applicability of prescribing medication by ICU nurses”, together with three sub-themes of “facilitators”, “potential risks of nurse prescribing” and “the professional pathway”, emerged. The use of successful global experiences, patient-oriented healthcare system policies, current culture and positive professional position of nurses, physician shortage, and high capacity of ICU nurses appeared as facilitators to perform the new role in our context. For the expansion of the new role, different professional pathways such as discussion with physicians and special groups with conflicts of interests, training qualified nurses in this area, and gradual development were proposed by the participants. The next step of the research is to prepare a set of standards for the prescription of medication by the ICU nurses in our context.

## Introduction

Nurse prescribing is a historic move in the world for nursing and an important part of the healthcare system solutions in the leading countries to facilitate patient’s access to medication [1], compensate for the lack of physicians’, and more optimal application of capabilities of nurses [2, 3]. Prescribing medication is part of the physicians’ role in the first place; however, nurses have officially and unofficially had a role in medication prescription during the past 50 years [4]. This concept was first proposed in 1969 in Idaho, USA and then paced a gradual and long way towards development. The number of countries where nurses have permits to prescribe is rapidly growing. Ladd *et al.* emphasized in their study that they were not looking for the answer to “Can nurses prescribe?”, but they were looking for “How developed is the role expansion currently?”. The relevant policies, legal, educational, and organizational conditions of medication prescription by nurses differ in different countries [5]. Practitioner nurses [6] with a master’s degree are allowed to prescribe in the USA, New Zealand, Canada, and Australia. Moreover, medication prescription is allowed for registered nurses (RNs) with a bachelor’s degree in England and Ireland [7, 8].

Developing the role of nurse prescribing due to their advanced performances is extremely important in Intensive Care Units (ICUs) [9]. The British Association of Critical Care Nurses (BACCN) described the requirements of nurse prescribing in a statement in 2009 to support nurse prescribers in ICUs [10]. Prescribing medication by Acute Care Nurse Practitioners [11] was first proposed in the United States in 1969, and then England, Canada, Australia, and New Zealand also proposed this concept. Nurse prescribing has been widely developed in these wards due to the clinical needs of patients [11]. Nurses working in ICUs are constantly preparing and using medication and are familiar with the complexities of prescription in the units [12]. Considering the high-risk medications used in this unit, the role of ICU nurses is essential [13]. However, the prescription of medications should be limited to qualified and experienced nurses [14].

Although this issue was noticed by different countries in past years [15, 16], such a permit has not been granted for nurses in the health system in Iran. However, a qualitative study showed that despite the lack of permission for nurse prescribing in Iran, it is unofficially taking place in hospital units, particularly in ICUs [14]. At the same time, nurses must perform within the competence framework of the rules [17]. A literature review showed that there is no evidence of the expansion of nurse prescribing roles in the healthcare system of Iran. As it is critical to start work on this new role of nurses in countries where nurse prescribing is not addressed yet [18], this study was done. The current qualitative study was conducted to explore the experiences of experts regarding the expansion of prescribing medication roles by the ICU nurses in our healthcare system.

## Material and Methods

### Study Design

This study is a qualitative research and part of a larger multi-method study. The study was carried out from April 2019 to February 2020.

### Setting and Participants

In this study, 30 participants were selected using purposive (19 individuals) and snowball (11 individuals) sampling methods. The data were collected through 31 individual semi-structured interviews. One of the participants was interviewed twice. The participants included nurses (10), physicians (5), and pharmacists (2), faculty members of the medical universities (5), nursing managers (4), and health policymakers at the Ministry of Health and Medical Education (the deputy of nursing and nursing committee board), and Iranian Nursing Organization (4).

### Data Collection and Analysis

The data were collected through semi-structured interviews. The interview time varied between 27–118 minutes, with an average of 56±19.3 minutes. Prior to each interview, the first author, in an informal meeting with the participants, obtained their informed written consent form to participate and record the interview. The time and place of the interviews were selected by the participants. The interviews continued until data saturation, up to the point where no new information was extracted. All interviews started with a general question on the familiarity with the concept of nurse prescribing. Some of the main questions included: “What is your experience or idea about legitimizing nurse prescribing for our ICU nurses in the present conditions of the healthcare system?”, “What barriers and facilitators do you think there are on the way of implementing this new role?”, “Please explain your experiences of prescribing medication to your patients or explain your colleagues’ experiences”. Throughout the study, exploratory and probing questions were also asked as follows: “Please explain more”, “Please give me an example to further clarify or understand the issue with an emphasis on the participants’ experiences”.

The data analysis was done by a conventional content analysis according to Graneheim and Lundman’s (2004) approach [19], using MAXQDA 10. The recorded data were carefully listened to by the first author and immediately upon each interview. Following this, data was transcribed word by word. Transcribed texts were compared with recorded interviews, and after ensuring accuracy, each interview was considered a unit of analysis. After multiple reviews and immersion in the data, the first author obtained a general understanding of the content. Then, the meaning units of each text were identified, and the initial codes were extracted. Initial codes with similar meanings were classified into broad subcategories and categories at the discretion of the research team (Table 1). Ultimately, subcategories formed the categories based on their commonalities.

**Table 1.** The results of qualitative data analysis, including main theme, categories, and subcategories.

**Theme**	**Categories**	**Subcategories**
**The applicability of prescribing medications by ICU nurses**	The facilitators of nurse prescribing (opportunities)	• Using successful global experiences; • Patient-oriented policy of the healthcare system; • The current culture and the positive professional position of nurses; • The shortage of physicians; • The high capacity of nursing.
	The potential risks of nurse prescribing (threats)	• Increased professional and legal responsibilities; • Obtaining authority by unqualified nurses; • The objections of physicians and special groups to the nurse prescribing role.
	The professional pathway	• Discussion with physicians and special groups with conflicts of interests; • Training qualified nurses; • Gradual development.

### The rigor of the study

The rigor of the study was evaluated using Lincoln and Guba’s criteria (2005), which included credibility, dependability, conformability, and transferability [20]. To assess data accuracy, the first author was constantly involved in the process of data collection and analysis and was completely immersed in it for several months. Moreover, the research team members constantly assessed all the results and discussed extracted themes, categories, subcategories, and codes. In order to ensure the reliability of the data, the extracted codes were evaluated by three participants. All analyses were audited by three external evaluators holding a doctoral degree in nursing. All the study steps were described in detail so that the other authors could repeat them in the future. Also, to reassure data transferability, the participants were selected with maximum variation in their age, gender, profession, level of education, organization, and work experiences. Moreover, data was obtained after immediate interview transcribing, accurate description of statements, objective experiences, and providing examples and quotations of the participants.

## Results

### Sample Characteristics

Data analysis from 30 participants and 31 interviews revealed that the mean age of the participants was 47.1±11.1 years. Moreover, the mean work experience and ICU experience were 21.1±11.2 and 12.2±7.3 years, respectively. In this study, 72% of the participants were males (n=22). The participants included 10 nurses, five specialists, two clinical pharmacologists, five faculty members from medical universities, four nursing managers, and four health policymakers.

The current qualitative study resulted in 2064 initial codes and 11 subcategories. The main theme was “the applicability of prescribing medications by ICU nurses”. The categories included “facilitators of nurse prescribing (opportunities)”, “potential risks of nurse prescribing (threats)”, and “the professional pathway”.

### Theme: The applicability of prescribing medications by ICU nurses

Our findings confirm the possibility of legalizing and implementing medication prescription by ICU nurses in the Iranian healthcare system.

#### Category 1: The facilitators of nurse prescribing (opportunities)

“Considering successful global experiences” and “inspiration from credible reports” were among the facilitators. All participants believed that implementing this role would be possible given the development and implementation of this role in many countries and their successful experiences through “considering the philosophy of leading countries”, “the framework of nurse prescribing in leading countries”, and “the reported outcomes of the effects”.

Participant 28, a policymaker, said, “*…this practice has become legal in many countries. They started based on need, wisdom, and science, and they observed that they could save the patient’s life because constant access to physicians is difficult, more expensive, and more costly. The situation in our country is the same*”.

According to the participants, the policy of the healthcare system is a priority in all health-oriented organizations. Therefore, a capable nursing system is an important asset to provide high-quality services, satisfy the needs of the people, and implement this policy.

“The current culture and the positive professional position of nurses” can also be helpful in this regard. “The nature of the relationship between the patients and their families and the nurses”, “better acceptance of the nursing position in recent years, increasing awareness of the society”, and “the demand for justice in obtaining health services” were among the subcategories. According to participants, nurses have the highest level of communication with people from the medical staff. According to them, nurses are in the front line of providing health services, and they are trusted by patients and their families. On the other hand, the professional position of nurses has improved in recent years. Moreover, due to increased health literacy, people have become more cautious, and the internet and social media played an important role in this regard. Consequently, medical malpractice and errors are now easily followed, and people are seeking legal remedies.

The shortage of physicians was another category. The country’s large population and the low number of physicians, especially in the underdeveloped and remote areas, create an opportunity to expand the role of nurses.

“*…Not only do we have a lack of available resources, but we also have a severe shortage of physicians according to international statistics, particularly specialist physicians in underdeveloped and remote areas…*” (Participant 21).

Moreover, the nature of the relationship between physicians and nurses is better and closer in ICUs than in other wards. According to the majority of the participants, most ICU physicians agree with implementing nurse prescriptions. The physicians trust and communicate with capable and experienced nurses they know. According to all the participants, cooperation from physicians is possible after achieving trust and understanding this policy. The majority of the participants believed that most ICU physicians agreed with nurse prescribing.

“*I prescribed medication in areas where I felt I could do it, the physicians that I worked with trusted me …*” (Participant14, nurse).

The nursing system is an important asset to provide high-quality services, satisfy the needs of the people, and implement this policy. The high capacity of nursing is the most important and tremendous asset to make the implementation of nurse prescribing feasible. Being equipped with a young, motivated, knowledgeable, and skilled nursing workforce, the presence of capable faculty members, the presence of credible educational centers, and different nursing higher education specialties were mentioned.

“*In fact, this is a good thing for our society. Our nurses are both educated and motivated, and they are constantly attracted by other countries! I have the documents*” (Participant 21, a health policymaker in the Iranian Nursing Organization).

“*…Our nurses have many abilities, such as professional conscience and ethics, professional science, humility …*” (Participant 28, hospital matron).

Moreover, a limited and controllable environment, continuous monitoring of patients with advanced equipment, fewer patients with a higher hospital stay, specialized functions, and better skills of ICU nurses lead to a better understanding of the patient’s clinical conditions. In addition, nurses working in this ward play an important role in the treatment decisions of the physicians. On the other hand, this issue was presented in previous sessions to the Deputy Minister of Nursing, Ministry of Health. The approval of nurses’ Clinical Competency License in recent years led to the specialization of the nursing profession while providing a ground for legalizing nurse prescribing.

“*I am very happy that you are researching this topic because we have discussed this issue before …*” (Participant 21).

#### Category 2: The potential risks of nurse prescribing (threats)

“Increased responsibility of nurses and legal consequences”, “obtaining authority by unqualified nurses”, and “the objections of physicians and special groups to the nurse prescribing role” were the subcategories mentioned by the majority of the participants. This is because nurse prescribing leads to a higher workload and increased professional, legal, and scientific responsibilities, and if neglected, it can increase the legal consequences and referrals to legal authorities.

“*A new duty is laid on nurses, and well, it will increase their responsibilities and accountability for their actions, and the complaints to legal authorities might increase*” (Participant 22).

Obtaining authority by unqualified nurses was the most important concern. This is because it may lead to wrong prescriptions and unethical actions. For example, false self-confidence among some nurses might lead to prescribing outside their field of expertise and without consulting the physician. Therefore, it would harm the identity of professional nursing, threaten the patients’ health, and increase per capita drug use following non-principled prescriptions.

“*Its biggest weakness is that nurses who are not really eligible might enter this field, or some of them may become too ambitious and prescribe unrelated medications*” (Participant 7, nurse).

The “objections of physicians and special groups to the nurse prescribing role” was the last subcategory that could lead to their non-cooperation. This objection could be due to lack of awareness, fear of nurses interfering with patients’ therapeutic protocol, and fear of harming their monopolization and financial interests due to the decreased number of visits.

During the interviews with physicians, three of them fully agreed with this proposal, while two disagreed, and none of the two accepted that this role was legalized in other countries, mainly due to their unawareness.

#### Category 3: The professional pathway

Participants believe that it is currently possible to implement this role. “Discussion with physicians and special groups with conflicts of interests” (such as physicians, pharmacologists, and insurance companies) is possible through gaining their trust, having scientific discussions, providing credible documents, and discussing the experiences of successful countries. Physicians’ participation in designing standards and quality control and using their expertise in selecting, controlling, and monitoring eligible nurses is also important.

“*Having conversations with those who have a conflict of interest, like physicians or insurance and policymaking companies...*” (Participant 23, a faculty member at the university).

Most of the participants believed that it would be better to have a gradual start in the initial steps of implementing nurse prescribing. Moreover, the permission to prescribe should be limited to several low-risk medications based on a specific protocol. This license is designed for emergencies to save patients’ lives and expand the nurses’ authority and increase the number of drugs they can prescribe over time. Furthermore, specialist nurses with higher education and several years of clinical experience should be prioritized for this role. In addition, it would be better to implement this as a pilot in an ICU with a few beds.

“*In the first stage, it is better to implement this as a pilot, select nurses based on the inclusion criteria, and assess their performance after granting the permission*” (Participant 16, a physician-fellowship).

## Discussion

This study was conducted to explore the experiences of experts about expanding the role of ICU nurses in prescribing medications in the Iranian healthcare system. Data analysis identified one theme that explains “the applicability of prescribing medications by ICU nurses” together with three sub-themes of “the facilitators”, “the potential risks of nurse prescribing”, and “the professional pathway”.

According to the participants, using successful global experiences, patient-oriented healthcare system policies, the current culture and positive professional position of nurses, the shortage of physicians, and the high capacity of ICU nurses were facilitators for the new role of nurses in our context.

During the recent decade, nurse prescribing has been considered in different countries, and the number of countries trying to implement and legalize it is increasing daily [15, 16]. Therefore, evaluating the philosophy behind the legalization of nurse prescribing in developed countries is highly important. The increased number of patients and their needs, the improved level of education among nurses, the increased workload of physicians, and the increased costs are among the reasons for legalizing this practice. Therefore, determining the threats and consequences of nurse prescribing in these countries can turn them into models for countries where this concept is still not legalized [21]. For example, British nurses hold the highest level of rights to prescribe medications globally; hence, this country is a successful model in implementing nurse prescribing [22, 23].

The current culture and positive professional position of nurses are other facilitators. Participants believed that patients and their families had a more comfortable relationship with nurses. Moreover, the professional position of nurses has improved in recent years. The relationship between patients and nurses is a key factor in the success of nurse prescribing [24]. Since nurses traditionally have more time to communicate with patients, they can be in a unique position to accurately assess the needs of the patients and the effects of the prescribed medications [25]. Today, people are more cautious and sensitive about their health and the service received due to their higher awareness and knowledge. Therefore, the patient-centered promotion of providing services is among the important policies in the healthcare system. Increasing awareness of the role of nurse prescribing has a significant effect on reducing sensitivity and creating a positive attitude in people. Many people do not know of this license in other countries, and even in countries where this practice is not legalized, nurses are allowed to prescribe medications to save patients in emergencies and the intensive care unit [18].

The shortage of physicians and the high capacity of ICU nurses were also mentioned during the interviews. Iran is a vast country with a large population, and the lack of specialist physicians, particularly in underprivileged and remote areas, is an opportunity for expanding the role of nurses. The lack of healthcare specialists is one of the main reasons for insufficient access to healthcare services [26].

High nursing capacity, *i.e.*, being equipped with a young, faithful, moral, motivated, knowledgeable, and skilled nursing workforce, is the most important asset in the healthcare system of Iran. According to the participants, the constant presence of nurses at the patients’ bedside would lead to higher familiarity and increase the possibility of providing direct and objective services to ICU patients. Given their continuous presence, nurses can monitor the quality of the care provided, and if necessary, they can consult with relevant physicians regarding patients’ conditions [27]. When prescribing medications, physicians make decisions not only based on their expertise and knowledge but also under the influence of nurses [28]. The nature of communications in ICUs is better than that of other units, and effective physician-nurse cooperation is necessary and should be strengthened for optimal treatment of patients [29]. This relationship is an appropriate facilitator, and nurses play a key role in creating interprofessional cooperation [30].

The potential risks of nurse prescribing constitute another highlighted category of this study. These risks included increased professional and legal responsibilities and the need for additional studying, which, if neglected, could lead to legal consequences for nurses. Prescribing medications by nurses complicates care provision and increases the need for interaction between physicians and nurses, accompanied by a significant increase in legal and professional responsibilities [31]. In a study, increased workload and responsibility, prescribing out of the field of expertise, and competency were reported as challenges experienced [32]. All participants highlighted concerns over obtaining authority by unqualified nurses. In addition to harming nurses’ reputations, this ineligibility could endanger patients’ safety. However, studies have addressed concerns over increased errors in prescribing medications by nurses and consequent dangers for patients as unnecessary concerns [33]. Physicians’ objection to the prescribing role of nurses was another threat. Our investigation reveals that this issue was experienced in other countries as well. The ambiguities of boundaries and the scope of action in nurse prescribing have been among the reasons for delaying the legalization of the practice [21]. Hierarchical norms and the top-down attitude of physicians can lead to a critical view of the issue, dissatisfaction, and lack of cooperation [21]. This is because they believe that expanding this role disrupts the hierarchical norms, based on which the physician is at the top of the decision-making hierarchy, while nurses are considered the executors of commands. This attitude has created unfair constraints and criticisms with regard to developing the role of prescribing medications by nurses [34].

In order to expand the new role, different professional pathways were proposed by the participants, such as having discussions with physicians and special groups with conflicts of interest, training qualified nurses in this area, and implementing gradual development. Participants emphasized physicians’ cooperation in designing standards and quality control and using their experiences to decrease objections. Ensuring the physicians and not replacing them with nurses, not interfering with their therapeutic decisions, and considering only the treatment of the patients, especially in cases of lack of access to physicians, can increase the cooperation of physicians.

Moreover, the participants mentioned the role of this issue in the health economy and cost-effectiveness, emphasizing people’s needs, conversations with insurance companies, holding meetings to interact with the beneficiaries and mutually interested groups, and convincing policymakers through increasing awareness and changing attitudes. The unfamiliarity with this issue is among the barriers to expanding this role [35]. The participants proposed that empowerment and training of qualified nurses were important to reduce these sensitivities.

It will be better to have a gradual development to successfully implement nurse prescribing in the ICU, such as granting a permit for a limited list of low-threat routine medications with a specific protocol related to emergency conditions to save patients. In this way, expanding duties and increasing the number of medications will be gradual. Moreover, specialist nurses with higher education and several years of clinical experience should be prioritized for this role. Results of different studies indicate that the process of granting permission for nurse prescribing has gradually developed.

Our study has some limitations that should be considered. First, the study took place in ICU, so there are limitations for generalizing our findings to other clinical wards. Thus, it is recommended that further studies be conducted in other settings.

Furthermore, although we tried to select participants with maximum diversity in age, sex, profession, level of education, place of work, and work experience in this study, we did not have the opportunity to interview health insurance managers and the organization of the medical system. In other studies, it is recommended to interview them individually or in focus groups.

## Conclusions

Our findings showed that, according to the participants, using successful global experiences, patient-oriented policies of the healthcare system, the current culture and positive professional position of nurses, the shortage of physicians, and the high capacity of ICU nurses were facilitators for implementing the prescription of medication by ICU nurses. However, there may be threats in the improper implementation. Increased professional and legal responsibilities of ICU nurses, the objections of physicians and other groups, and obtaining authority by unqualified nurses are among these threats. Professional pathways, such as discussion with physicians and special groups with conflicts of interests, training qualified nurses, and gradual development, were proposed by the participants. However, health policymakers are expected to take steps to minimize potential threats in the process of role expansion. Future research has been planned to prepare a set of standards for prescribing medication by the ICU nurses in our context.

Several clinical implications are evident from this study. Given the lack of physicians and financial resources, Iran’s diverse population, and patients’ needs, the quality-of-care services in ICUs could be improved by expanding the role of nurses in prescribing medication.

The legality of nurse prescribing in the ICUs can prevent medication errors and endangering patient safety and protect nurses from the consequences of illegal prescriptions in emergencies in ICUs.

## Acknowledgments

### Conflict of interest

The authors declare no conflict of interest.

### Ethical approval

This study was approved by the Research Ethics Committee of Shahid Beheshti Medical University in Tehran (Code: IR.SBMU.PHARMACY.REC.1398.023).

### Consent to participate

Written informed consent was obtained from all the participants.

### Data availability

The datasets analyzed during the current study are available from the first author on reasonable request.

### Personal thanks

The research team sincerely thanks all those who contributed to this study and participated in the interviews.

### Authorship

AN, AA, MP, CR, and RJ contributed to designing the study. AN collected the data and performed data analysis. The final report and manuscript were written by AN, AA, MP, CR, and RJ. All the authors read and approved the version for submission.
